# Protein-bound polysaccharide K suppresses tumor fibrosis in gastric cancer by inhibiting the TGF-β signaling pathway

**DOI:** 10.3892/or.2014.3636

**Published:** 2014-11-28

**Authors:** TOSHIFUMI SHINBO, SACHIO FUSHIDA, TOMOYA TSUKADA, SHINICHI HARADA, JUN KINOSHITA, KATSUNOBU OYAMA, KOICHI OKAMOTO, ITASU NINOMIYA, HIROYUKI TAKAMURA, HIROHISA KITAGAWA, TAKESHI FUJIMURA, MASAKAZU YASHIRO, KOUSEI HIRAKAWA, TETSUO OHTA

**Affiliations:** 1 Department of Gastroenterological Surgery, Division of Cancer Medicine, Graduate School of Medical Science, Kanazawa University, Kanazawa 920-8641, Japan; 2 Center for Biomedical Research and Education, School of Medicine, Kanazawa University, Kanazawa 920-8641, Japan; 3 Department of Surgical Oncology, Osaka City University, Graduate School of Medicine, Abeno-ku, Osaka 545-8585, Japan

**Keywords:** protein-bound polysaccharide, transforming growth factor β, gastric cancer, cancer-associated fibroblast

## Abstract

Peritoneal carcinomatosis (PC) is the most frequent metastatic pattern of gastric cancer and its prognosis is extremely poor. PC is characterized by rich fibrosis and the development of obstructive disorders such as ileus, jaundice and hydronephrosis. Epithelial-mesenchymal transition (EMT) is one of the major causes of tissue fibrosis and transforming growth factor β (TGF-β) has a pivotal function in the progression of EMT. Protein-bound polysaccharide K (PSK) is a biological response modifier that can modulate the TGF-β/Smad signaling pathway *in vitro*. In the present study, we established a fibrotic tumor model using human peritoneal mesothelial cells (HPMCs) and a human gastric cancer cell line to evaluate whether PSK attenuates tumor fibrosis. HPMCs exposed to PSK did not undergo the morphological change from a cobblestone-like pattern to a spindle-shape pattern normally induced by treatment with TGF-β. Immunofluorescence further demonstrated that PSK suppressed TGF-β-induced overexpression of α-SMA in the HPMCs. We further showed that HPMCs contributed to the proliferation of tumor fibrosis by using a mouse xenograft model. Additionally, PSK treatment of these mice significantly reduced the area of observable tumor fibrosis. These results suggest that seeded cancer cells transformed HPMCs into myofibroblast-like cells through their release of TGF-β in the microenvironment, facilitating the development of fibrous tumors in organs covered with HPMCs. Therefore, our study indicates that PSK has potential utility as an anti-fibrotic agent in the treatment of gastric cancer patients with PC.

## Introduction

Gastric cancer is a major global health concern, with an estimated 989,600 new cases and more than 738,000 attributable deaths in 2011 (1). Peritoneal carcinomatosis (PC) is a characteristic feature of gastric cancer and is a critical factor underlying its poor prognosis (2–4).

PC is relatively resistant to systemic chemotherapy due to the poor blood supply and oxygenation of cancer cells in the peritoneum (5). A large body of data has shown that systemic administration of S-1 or taxanes may be efficacious in treating PC. These compounds have a high sensitivity in targeting poorly differentiated adenocarcinoma, which is a common microscopic type of peritoneal tumor (6,7). Furthermore, when administered intravenously, some of these compounds are transported into the peritoneal cavity (8,9). Therefore, combining S-1 with intraperitoneal (i.p.) administration of taxanes has been proposed as an effective and feasible treatment strategy for PC. Ishigami *et al* established a protocol of i.p. paclitaxel with S-1 plus intravenous paclitaxel, in which the median survival time (MST) was 22.5 months and the 1-year survival rate was 78% (10). S-1 plus i.p. docetaxel also demonstrated a promising 1-year survival rate of 70%, with an MST of 16.2 months in patients with severe PC (11). However, these clinical outcomes have been largely unsatisfactory since most patients die of recurrence within 5 years. PC is characterized by cancer cell infiltration and proliferation, which is accompanied by extensive stromal fibrosis. This results in the development of chemoresistance and obstructive disorders such as ileus, obstructive jaundice and hydronephrosis (12). Therefore, it is necessary to develop new treatment strategies to target the fibrosis in PC.

The fibrous tissue found in organs during PC is produced by cancer-associated fibroblasts (CAFs), which are recruited from orthotopic fibroblast pools (13), bone marrow-derived fibrocytes (14), and human peritoneal mesothelial cells (HPMCs) (15). These cells can undergo epithelial-mesenchymal transition (EMT) to differentiate into an extracellular matrix-producing myofibroblastic phenotype in the presence of TGF-β released from gastric cancer cells (16). Therefore, TGF-β signaling represents a promising potential target for tumor fibrosis in PC.

Protein-bound polysaccharide K (PSK; Krestin^®^) is isolated and purified from the cultured mycelium of the Basidiomycete *Coriolus versicolor* (17). PSK is considered a biological response modifier, and has been approved for use in combination with chemotherapy to prolong the survival of patients with gastric cancer or colorectal cancer.

PSK also appears to inhibit TGF-β signaling through suppression of TGF-β production, direct binding with TGF-β, and through acting on TGF-β receptors (18–20). Ono *et al* further reported that PSK can suppress Smad2 phosphorylation, resulting in the inhibition of EMT in the colorectal cancer SW837 cell line (21). Here, we investigated whether PSK could inhibit both the EMT-like change of HPMCs in response to TGF-β signaling *in vitro*, and the subsequent induction of tumor fibrosis by co-inoculum of gastric cancer OCUM-2MD3 cells and HPMCs *in vivo*.

## Materials and methods

### Cell lines and cell culture

HPMCs were isolated from surgical specimens of the human omentum, as previously described (22). Written informed consent for use of these specimens, as required by the Institutional Review Board at Kanazawa University, Japan, was obtained from patients undergoing elective abdominal surgery. Small pieces of omentum were immediately washed extensively in phosphate-buffered saline (PBS) and were incubated in pre-warmed PBS containing 0.125% trypsin/EDTA (Gibco/Invitrogen, USA) for 30 min at 37°C. The suspension was then passed through a 100-μm pore nylon mesh (Becton-Dickinson, Japan) to remove undigested fragments and centrifuged at 1,500 rpm for 5 min. The collected cells were cultured in RPMI-1640 medium (Gibco/Invitrogen) supplemented with 20% heat-inactivated fetal bovine serum (FBS; Nichirei Bioscience Inc., Japan), 100 IU/ml penicillin, 100 mg/ml streptomycin (Gibco/Invitrogen), and 2 mM glutamine (Nissui Pharmaceutical Co. Ltd., Japan). The cells were seeded in gelatin-coated 75-cm^2^ flasks (BD BioCoat, USA) and cultured in 10 ml of medium at 37°C in a humidified atmosphere of 5% CO_2_ in air. Subconfluent HPMCs were trypsinized with 0.125% trypsin/EDTA before use. HPMCs were used from passage 1 to 3 in all experiments.

OCUM-2MD3, a cell line derived from a human scirrhous gastric cancer and with high peritoneal-seeding activity, was kindly provided by the Department of Surgical Oncology of Osaka City University of Medicine. OCUM-2MD3 cells were seeded in 75-cm^2^ dishes (Becton Dickinson, Tokyo, Japan) and cultured in 10 ml Dulbecco’s modified Eagle’s medium (Life Technologies, Tokyo, Japan) supplemented with 10% heat-inactivated FBS, 100 IU/ml penicillin, 100 mg/ml streptomycin, 2 mM glutamine, and 0.5 mM sodium pyruvate, at 37°C in a humidified atmosphere of 5% CO_2_ in air.

### Reagents

Protein-bound polysaccharide was kindly provided by the Kureha Chemical Ind. Co. (Japan) and TGF-β was purchased from Sigma-Aldrich, Inc. (USA).

### Phase contrast microscopy

HPMCs in cultures were treated with TGF-β1 (10 ng/ml) or both TGF-β1 and PSK (100, 500 μg/ml) for 72 h and morphological changes were visualized by phase contrast microscopy. The images were captured using a Nikon inverted microscope (Nikon Corp., Japan).

### Immunofluorescence

For visualization of E-cadherin and α-SMA in the HPMCs, the cells were grown on 4-well, collagen type I-coated culture slides (BD BioCoat) and then fixed in a 1:1 mixture of methanol and acetone for 10 min. Briefly, the slides were immersed in methanol containing 0.3% H_2_O_2_ for 30 min, blocked with 3.3% normal goat serum in PBS, and incubated with the E-cadherin antibody (H-108, rabbit polyclonal IgG; diluted 1:100; Santa Cruz Biotechnology, Inc., USA) and α-SMA (1A4, mouse monoclonal IgG; diluted 1:100; DakoCytomation, Denmark) at 4°C overnight. Following three PBS washes, immunoreactivity was visualized by incubating the sections with an anti-mouse IgG antibody conjugated with Alexa Fluor^®^ 488 and an anti-rabbit IgG antibody conjugated with Alexa Fluor^®^ 546 (1:400; Molecular Probes/Invitrogen, USA) for 1 h at room temperature. Cells were then incubated with Hoechst 33258 for nuclear staining for 5 min and mounted with propyl gallate containing phenylenediamine under glass coverslips. Slides were observed with an immunofluorescence microscope (BX50/BX-FLA; Olympus, Japan).

### Mouse xenograft model

All animal experiments were performed according to Kanazawa University’s standard guidelines. Female immunocompromised BALB/c-*nu/nu* mice (4–6 weeks old; Charles River Laboratories Inc., Japan) with an average body weight of 20 g were maintained under sterile conditions and used for all *in vivo* experiments. OCUM-2MD3 cells were co-cultured with an equivalent number of HPMCs, and a total of 5×10^6^ cells in 100 μl of RPMI-1640 was then subcutaneously injected into the dorsal side of each mouse on day 0. Mice were then divided into two groups: i) mice given normal chow (control, n=10) and ii) mice given chow mixed with 1% PSK from day 1 (PSK, n=10). The PSK concentration within the chow was adjusted to be at a dose of approximately 1 g/kg body weight/day, which was 1.5-fold higher than the clinical dose of PSK (3 g/day) estimated by the surface area normalization method (23). Mice were allowed unrestricted access to water and to the standard or mixed chow. On day 15, the animals were sacrificed, and the tumors were harvested. Tumor specimens were collected for immunohistochemical examination.

### Histological and immunohistochemical examination

Tumor specimens were fixed in 10% neutral-buffered formalin and embedded in paraffin. Sections were stained with hematoxylin and eosin and Azan. To analyze fibrosis, Azan (blue)-stained areas were measured on a video display (magnification, ×200) in a blinded manner using a QuickGrain digital image analyzer (Inotech, Hiroshima, Japan). Two sections were selected randomly from each sample and three fields from each section were evaluated; samples from 10 mice in each group were examined. To evaluate α-SMA expression, the sections were immunostained with an α-SMA antibody (1A4, mouse monoclonal IgG, diluted 1:100; DakoCytomation, Japan) at 4°C overnight, and treated with EnVision reagent (Dako Co., Japan) for visualization.

### Statistical analysis

All data are expressed as mean ± SD. Statistical analyses were conducted using SPSS statistical software, version 11.0 (SPSS, Inc., USA). Comparisons of drug effects were carried out using the Student’s t-test. A p-value of <0.05 was considered to indicate a statistically significant difference.

## Results

### Effect of PSK on the morphological change in HPMCs following treatment with TGF-β1

**Figure 1:**
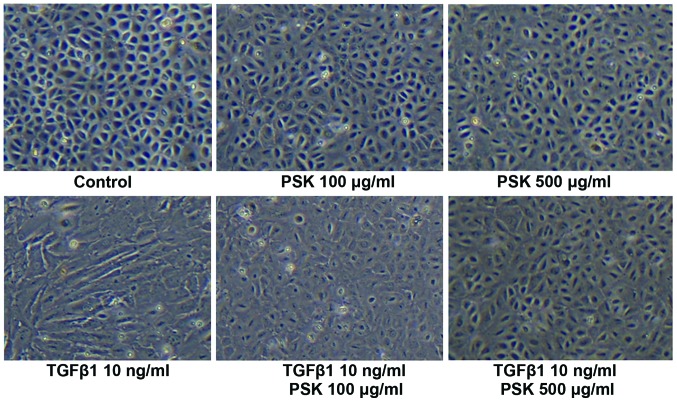
Representative image of morphological changes in HPMCs. (Upper left) HPMCs cultured in control medium. (Upper middle) HPMCs cultured in medium containing 100 μg/ml of PSK. (Upper right) HPMCs cultured in medium containing 500 μg/ml of PSK. (Lower left) HPMCs cultured in medium containing 10 ng/ml of TGF-β. (Lower middle) HPMCs cultured in 10 ng/ml of TGF-β and 100 μg/ml of PSK. (Lower right) HPMCs cultured in 10 ng/ml of TGF-β and 500 μg/ml of PSK. HPMCs cultured in each condition for 72 h were visualized by phase contrast microscopy at magnification, ×200. HPMCs, human peritoneal mesothelial cells; PSK, protein-bound polysaccharide K.

Control HPMCs exhibited a polygonal and cobblestone-like growth pattern, whereas HPMCs treated with TGF-β1 adopted the spindle-shaped morphological characteristic of fibroblasts. Pretreatment with PSK blocked these morphological changes induced by TGF-β1 in a concentration-dependent manner (Fig. 1).

### Immunofluorescence examination

**Figure 2:**
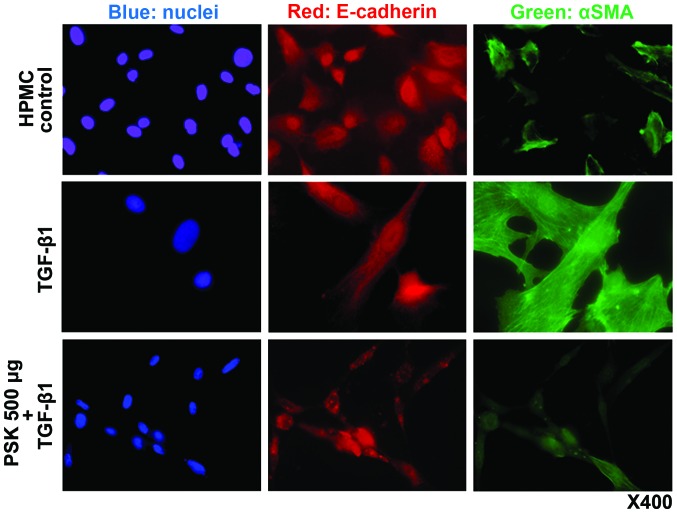
Representative photomicrographs of HPMCs, labeled with antibodies to E-cadherin (red) and α-SMA (green). TGF-β-treated HPMCs showed increased expression of α-SMA, whereas both TGF-β and PSK-treated HPMCs showed almost equal expression of α-SMA when compared with the control. HPMCs, human peritoneal mesothelial cells; PSK, protein-bound polysaccharide K.

Expression of E-cadherin and α-SMA were evaluated by indirect immunostaining and confocal microscopy. In the absence of TGF-β1, HPMCs did not express α-SMA in the cytoplasm. Treatment with TGF-β1 induced cytoplasmic α-SMA expression, a recognized component of EMT change. Pretreatment of HPMCs with PSK prior to TGF-β1 administration prevented this EMT-like change (Fig. 2).

### Histological and immunohistochemical examination of the xenograft tumors

**Figure 3:**
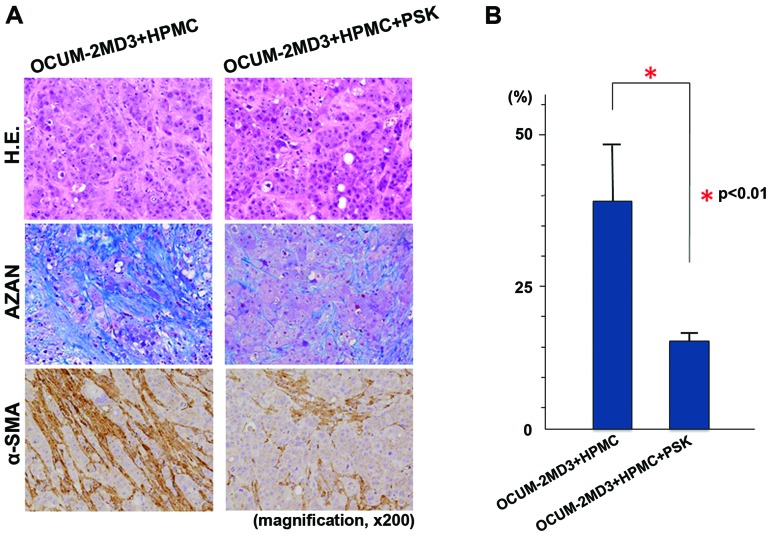
(A) Macroscopic views of mouse xenograft tumors. (Left panels) Xenograft tumors inoculated with OCUM-2MD3 cells and HPMCs contained much fibrous stroma showing blue color by Azan staining and many α-SMA-positive cells. (Right panels) Subcutaneous tumors in mice treated with PSK revealed little fibrous stroma and a small number of α-SMA-positive cells. (B) The fibrous area was measured semi-quantitatively. The PSK treatment group showed a significant low ratio of fibrosis when compared with the control (PSK not treated) group (p<0.01). HPMCs, human peritoneal mesothelial cells; PSK, protein-bound polysaccharide K; H.E. hematoxylin and eosin.

Xenograft tumors resulted from the inoculation of BALB/c-*nu/nu* mice with a suspension of OCUM-2MD3 cells and HPMCs contained many α-SMA-positive cells and a large amount of collagen fibers. Administration of PSK to these mice yielded a reduction in the number of α-SMA-positive cells and a decreased amount of collagen fibers (Fig. 3A). To semi-quantitatively evaluate the degree of tumor fibrosis, we examined the fibrotic areas in each tumor sample. Azan staining revealed a significant difference in the degree of fibrosis in the PSK group compared with the control (p<0.01; Fig. 3B).

## Discussion

The source of CAFs has not been fully elucidated. However, orthotopic fibroblasts and bone marrow-derived cells may function as myofibroblasts during EMT. In line with these findings, we previously reported that bone marrow-derived fibrocytes can contribute to tumor proliferation and fibrosis in gastric cancer (14).

In this present study, we demonstrated that HPMCs transformed into myofibroblast-like cells following exposure to TGF-β, and that these cells contributed to tumor-associated fibrosis. We previously demonstrated that HPMCs promoted fibrosis in a mouse xenograft model when co-inoculated with MKN45 cells (15). Together, these results suggest that HPMCs have a latent ability to function as CAFs and induce fibrosis in the tumor microenvironment in multiple cancer cell types.

Although orthotopic cancer-associated fibroblasts cross-talk with gastric cancer cells to enhance tumor progression, free cancer cells in the intraperitoneal cavity could produce peritoneal fibrous tumors in the absence of orthotopic fibroblasts. In PC of gastric cancer, seeded cancer cells attach to HPMCs and transform them into myofibroblast-like cells by releasing TGF-β. Spindle-shaped HPMCs can then facilitate adhesion of cancer cells to the basement membrane and subsequently infiltrate the basement membrane as CAFs together with cancer cells. This concept is in agreement with the fact that PC can develop in any organ covered by HPMCs.

TGF-β is regarded as one of the key molecules responsible for the differentiation of a variety of precursor cells to a myofibroblastic phenotype (24). Our results revealed that HPMCs also adopt an elongated spindle-shaped morphology from their normal cobblestone-like growth pattern, and exhibit overexpression of α-SMA when exposed to TGF-β. This supports the notion that TGF-β secreted from OCUM-2MD3 cells transformed HPMCs into myofibroblasts within the microenvironment of our co-inoculated mouse model, leading to the promotion of tumors with a fibrous stroma.

The control of TGF-β signaling is required for the inhibition of organ fibrosis during PC, and for decreasing cancer invasiveness and metastasis. PSK is known to suppress TGF-β signal transduction through inhibition of Smad2 phosphorylation (21). We now demonstrated that PSK inhibited TGF-β-induced EMT-like change in HPMCs, and that fibrosis in xenograft tumors of PSK-treated mice was significantly reduced. Previous reports have shown that a TGF-β neutralizing antibody and a TGF-β receptor kinase inhibitor can both suppress EMT and reduce stromal fibrosis (25,26). However, the long-term clinical use of these agents may lead to major complications due to the likelihood of adverse effects from interference with the many important roles of TGF-β in normal tissues (27).

Previously, PSK has been thought to contribute to the maintenance of nutrition and immune strength in cancer patients during chemotherapy. As such, PSK has been approved for use in combination with chemotherapy to prolong the survival of patients with gastric or colorectal cancer. The effectiveness of PSK as a postoperative adjuvant for immunochemotherapy has been demonstrated by a meta-analysis (28). Here, the hazard ratio of 5-year survival was 0.88 (95% confidence interval: 0.79–0.98, p=0.018), verifying that chemotherapy + PSK enhanced the survival of patients with curatively resected gastric cancer. These randomized clinical trials were conducted to examine the effectiveness of postoperative adjuvant immunochemotherapy compared with chemotherapy alone.

At present, S-1 is the standard agent for adjuvant chemotherapy for stage II and III patients with curative resected gastric cancer in Japan (29,30). The Hokuriku-Kinki Immunochemotherapy Study Group on Gastric Cancer is undertaking a clinical trial of S-1 alone versus S-1 plus PSK for curatively resected stage II and IIIA gastric cancer (31). It is expected that the S-1 plus PSK group will demonstrate longer survival times than the S-1 alone group in those patients with peritoneal recurrence.

To date, PSK has been widely used clinically in combination with chemotherapy without the observation of any serious side-effects. Therefore, it may hold promise as an anti-fibrotic agent for the treatment of gastric cancer patients with PC.
